# Cognitive Domain Impairments Related to Walking Difficulty: A Harmonized Cognitive Assessment Protocol Study

**DOI:** 10.1093/geroni/igaf122.785

**Published:** 2025-12-31

**Authors:** Matthew Miller, Pamela Ygrubay, Irena Cenzer, Deborah Barnes, Katherine Possin, Kenneth Covinsky

**Affiliations:** University of California, San Francisco, San Francisco, California, United States; University of California, San Francisco, San Francisco, California, United States; University of California, San Francisco, San Francisco, California, United States; University of California, San Francisco, San Francisco, California, United States; University of California, San Francisco, San Francisco, California, United States; University of California, San Francisco, San Francisco, California, United States

## Abstract

Community-dwelling older adults with cognitive impairment are at risk of disability. This study aimed to identify if adding specific cognitive domain impairments increase the odds of walking difficulty beyond single domain impairments. We analyzed data from 3,374 community-dwelling older adults (mean[SD] age: 74.1[7.6] yrs, 55.5% female, 80.5% non-Hispanic White) in the Health and Retirement Study Harmonized Cognitive Assessment Protocol, a data-source for cognitive aging research. A validated algorithm was used to classify cognitive domain impairment for executive function (EXF), memory (MEM), language fluency (LFL), visuospatial (VIS), and orientation (ORI). Difficulty walking (≤1 block) was identified using participants’ self-report. We first calculated the prevalence of difficulty walking for each domain impairment. Next, we estimated age, sex, and race/ethnicity-adjusted odds of difficulty walking (aOR[95%CI]) for each domain impairment alone, and then in the presence of another domain impairment. The prevalence of difficulty walking was highest for EXF impairment (27.2%), followed by ORI (25.5%), VIS (19.1%), MEM (14.8%), and LFL (10.4%), relative to 13.6% without any domain impairment. Compared to no impairment, the odds of difficulty walking were greater with EXF impairment (2.23 [95%CI:1.41,3.52]), but no other single domain impairments (P > 0.05). EXF impairment increased the odds of difficulty walking beyond MEM (MEM+EXF:3.65[95%CI:1.36-9.84] and LFL (LFL+EXF: 8.57[95%CI:2.26-32.56]), but no other dual-domain impairments increased the odds above referent EXF, MEM, LFL, VIS, ORI impairments alone. To enhance clinical outcomes, further research must examine the consistency of relationships of EXF impairment, alone and with other domains, across meaningful measures of older adult function.

